# Chronic Abscess and Attendant Necrosis of the Superior Maxilla

**Published:** 1859-10

**Authors:** J. Carroll House

**Affiliations:** Lowville, N. Y.


### ARTICLE II.

*Chronic Abscess and Attendant Necrosis of the Superior Maxilla.* Case reported for the Journal, by J. CARROLL HOUSE, Lowville, N. Y.

ON the 5th of March, last, (1859,) Mr. Wm. R. G. called at my office to consult me in regard to a diseased mouth with which he said he had for a long time been afflicted. Previous to the examination, he gave me a brief outline of the history of his case, which, at a subsequent visit, I more particularly elucidated as follows. (I have taken the liberty to transfer his own language so far as I am able.)

"On the 20th of last May, 1858, I was taken with a very severe pain in the left side of my upper jaw, of a sharp darting, or piercing character, which increased until it extended to my whole head, causing my head to feel *as though there were a lot of cog-wheels in motion therein.* I made use of all the remedies which either myself or my friends could suggest." (Such as counter-irritants, fomentation, etc.) "The second day I was so unwell as to be confined to my bed a good share of the time.

"After enduring severe torture for a week, a neighboring physician, Dr. H., was called, who pronounced my affection an aggravated attack of neuralgia and ague of the face. He treated my case accordingly for some time. The roof of my mouth being badly swollen, he lanced it four or five times, but nothing but a dark bloody matter came from it. He managed to drive the pain from my head, but did not appear to affect the real seat of the disease materially.

"After treating me a while, he proposed that the right eye-tooth be extracted, to which I consented, although for aught I know it was perfectly sound and was not more tender than the rest. Following the extraction of which, there was discharged a small quantity of bad smelling yellowish matter, but the acute pain still continuing, and my general health becoming quite impaired, I concluded to apply to Dr. B., of this place. At this time I was also troubled with a dripping in the back part of my mouth, (from behind the velum pendulum palati,) which caused considerable irritation in my throat, with some cough.

"Dr. B. informed me that my complaint was a chronic affection of the glands of the mouth (?) implicating the gums, and that if the discharge was not soon stopped, it would lead to serious disease of the lungs and consequent death; but he felt confident he could cure me, to use his own words, 'me fix you sumting make you cum petter right off.' He gave me a stick of something about the size of a pipe-stem, (probably nit. sil.,) a piece of which I was to dissolve in water, and wash my gums with the same two or three times a day, and this would most certainly cure me in a very short time. This I did faithfully for some time, but continued to grow worse, and I thought most certainly I must die. The pain in my jaw and head had continued without cessation for some two months after the first attack, after which it would have intervals of recurrence.

Becoming quite alarmed about myself, and receiving no benefit from Dr. B's treatment, I was persuaded to consult Dr. F., of D. K., who pronounced my disease a case of alveolar abscess, and recommended the extraction of the left eye-tooth, which he did, and as he threw it directly into the stove, I had no chance to see whether it was decayed or not, but think not. Not long after this I began to have a constant discharge from the left nostril, and the opening which the extraction of the tooth just mentioned left began to increase in size and form a channel through which there was a constant discharge of matter." (The pain at this time he described as being of a dull, heavy, throbbing character, not so intensely excruciating as at first, but still extremely reducing.)

"Continued with Dr. F. until sometime in the winter, but he not being able to help me, I was led to seek the advice of Dr. A. who has been prescribing for me for some two months, but as I do not get any better, being advised by some friends, and thinking myself that it might be possible for a dentist to know more about the disease with which I am affected than physicians, I have come to consult you, and I want you to examine my mouth and see what *you* think of it."*

*Diagnosis.* Æ. 36. Temperament nervo-lymphatic, with a present extremely sensitive condition of the nervous; evident scorbutic or scrofulous tendency; manifest complication in the disease, as involving lungs and air passages, mucous membrane of throat, with a deranged stomach, though no great stomatitis, (as might naturally be expected in such complication.) Teeth in superior maxilla, normal, with the exception of canines, also first molar on left side, which were gone. The socket of the left canine tooth was the seat of a fistulous opening, through which was evacuated a fetid and sanious pus.

The mucous membrane of the palatine arch was very much distended and swollen, the line of distinction extending from the right lateral incisor back in a direct line to the posterior part of the arch, thence laterally to the right molar, and resembling in its convexity the shape of the half of an almond shell. The swelling was dense and firm, not at all fluctuating, with a well defined cicatrix of about five-eighths of an inch, extending in a line parallel

* I had at first thought that I would not insert the patient's own statement of his treatment previous to coming under my own, and content myself with giving my own diagnosis and treatment. But after considerations led me to believe that the merits of the case seemed peculiarly to demand that I should give my minutes *in full*. Yet in so doing I hope that I am actuated by the same spirit which the patient manifested in relating the details of his previous condition and treatment to me, viz. a sincere desire that his true condition might be fully comprehended and understood, without in the least degree casting any unkind or unjust reflections upon the character of his former medical advisers or their treatment, as none such were entertained, being all well known to me as standing well in their profession and their character above suspicion.

with the palatum-raphae, a line and a half to the right. In the space once occupied by the right canine, the gums appeared healthy, and the ridge of the jaw firm. There was a very disagreeable discharge from the left nasal fossa; though not abundant, there were also a couple of sinous openings, one on each side, in the depressions of the gums between the central and lateral incisors, two lines from the margin of the same. Upon injecting a stream of tepid water from a half oz. syringe, the pipe of which was introduced into the opening of the left canine socket, the injection found its way into the left nasal fossa, and also the two sinuses just mentioned in the front of the gums—not broken as from a ragged pipe, but a smooth jet, and perfect, thus showing that the sinuses had become perfectly formed.

The left lateral incisor adjoining the fistulous opening was slightly loose, the remaining incisors tolerably firm. The left bicuspids were easily moved in their places. A thin, slender probe, thrust into the fistulous opening, could be made to traverse two-thirds the distance toward the posterior margin of the palatine plate, yet without yielding to the sense of touch, any indications of present necrosis, such as a feathery or roughened condition of the lamina, still leaving no doubt but that the mucous membrane was seperated from the palatine plates, and also indicating a wasting away of the alveolar plates in the region of the fistula.

After examining the case as carefully as I was able, from the pressure of other engagements, and forming in my mind what I deemed a correct conclusion, from the above diagnosis, I informed the patient that it was my opinion that he was the victim of a most decided and aggravated case of carious ulceration, and possibly *necrosis* of the upper jaw, and it was quite probable that ere the ravages of the disease were stayed he would be obliged to submit to the loss of all the teeth which were loose, and possibly *all in the upper jaw.*

The patient seemed very desirous (naturally enough) that, *if it were a possible thing*, his front teeth might be preserved, to which I could give him no definite encouragement. He wished that I would make a prescription with a view to eradicate the disease, and at the same time save the front teeth.

*Treatment.* With a view of reducing the constitutional proclivity to scrofulous derangement, and thus get the system into a condition to assist a naturally healthy development among the diseased parts, when such should be brought about, I advised "chlo. potassa, one half drachm daily in half wine glass of water—stomach empty;" also gave as a caustic injection for the abscess, "nit. sil. 10 gr. cryst. to oz. aqua," the injection to be preceded by a thorough cleansing of the same with tepid water, following the caustic with a detergent and mild astringent injection from the following formula, slightly altered from Harris:

| | | |
|---|---|---|
| ℞ | Pul. nut gall, ʒ ij., | Infuse and filtra. |
| | Orris root, ʒ i., | |
| | Cort. cinchonæ, ʒ ij., | |
| | Aqua font, ℥ iv., | |

The caustic to be used once in two days; the detergent to be injected (after cleansing) three or four times in the day; cautioned the patient against the use of any fatty or heating food, advising a low and regular diet, avoiding exposure to changing air, lest the cold should induce local inflammation; with the order to call again at his earliest convenience. (Patient resides some considerable distance in the country, for which reason I shall not be able to see him as often as I could wish.)

March 22d. Symptoms not so aggravated, the exudations from fistula not so fetid or of so sanious a character, the pain not so intense or so oft recurring, otherwise the case remains the same as at first examination. Continue the treatment as above, cautioning the patient against entertaining too strong hopes.

April 2d. The discharge into the nose *entirely stopped*, the palatine swelling much reduced, pain recurring only at long intervals, (comparatively,) mucous membrane apparently healthy. The left bicuspids are quite loose, extract them; in so doing, develop the fact that the whole alveolus enveloping their fangs was not firm in its attachment to the jaw, or in a healthy condition, inasmuch as it could be moved by the motion of the tooth in extraction, although the remaining molar on this side is perfectly firm and healthy; expect the exfoliation of these alveolar pieces soon. On introducing a thin silver probe into the fistulous opening of the eye tooth, and thrusting it in the direction of the alveolar ridge to the right, (of patient,) the fangs of the front teeth could readily be distinguished, thus showing that the bony support at the back of these teeth had been destroyed by the carious abscess, but could not yet get the patient to consent to their extraction, although standing behind and above the patient, with the forefinger and thumb of each hand grasping the front teeth, they could be moved bodily "out and in," *and this without any pain to the patient.* (?)*

* Could the consent of the patient have been obtained, I had from careful reflection, decided upon an operation as follows:

Having administered the anæsthetic, giving the head of the patient in the charge of an assistant, who holds the upper lip retracted vertically against the nose; with a thin scalpel I make an incision to the right of the right lateral incisor, extending vertically from the margin of the gums about four lines, extending the cut in a posterior direction from the back of the tooth about the same distance, then dissect the gums clear from their margins, both buccal and lingual, to the fistulous opening, i. e., of left canine; turn back the flaps thus formed, and extract, in a mass, the four incisors with the alveolus which might be attached, (which, I doubt not, was entirely disconnected from the jaw,) then with a pair of sharp dissecting scissors trim neatly the margin of the flaps, bring them to their places, adjust them, and retain with sutures, (interrupted.) This operation, I have not the least doubt, would have been entirely successful, serving to hasten the patient's recovery, affording not only a chance to eradicate all of the diseased bone, but at the same time allowing of a careful and thorough examination of the jaw proper.

Discontinued the chlo. pot., continued the nit. sil. injection, and changed the detergent to

Tannicum purum—sol., ℥ iv.

Orris root, ℥ i.

Cort. cinchonæ, ℥ ij.

Aqua font oz. iv.

Tinct. arnica, oz. ½.

to be used as before; with a wash for the gums of chlo. pota., sol.

Case continued to improve, with exfoliations of the alveolus on left side, until May 12th, at which time a small speculum of bone manifested itself immediately over the *right* lateral incisor. Opening the gums with a horizontal thrust of the lance, I was enabled to introduce the beaks of a pair of bone tweezers, and with them move the bone, which, upon examination, showed itself to be the alveolus lying between the lateral and central incisor, as it had concavities on either side corresponding to the fangs of these teeth.

Yet, notwithstanding this continued loss of bony support, the front teeth seemed to remain remarkably firm, (comparatively speaking,) and appeared *even more so than at last record*, but predicate no encouragement to hope to save them, *as it would be preposterous.* The alveolus of the left side having exfoliated, the gums are healing kindly, and the swelling in the palatine arch is now confined to the anterior portion. The sanious discharge is changing to a more healthy pus. The patient has complained of but very little pain during the last month; the margin of the gums around the front teeth are quite natural, no sloughing or festooning, such as generally attend alveolar disease, and we are obliged to confess that all things seem to work together for good. Recommenced the chlo. potassa powders, and substitute a weak tincture of arnica injection, in place of the caustic and lotion.

May 28th. The two lateral incisors quite loose; the central incisors somewhat so; gums inflamed from cold. From the place whence the speculum of alveolus was eradicated, over right incisor, (see last entry,) was now exuding a discharge akin to healthy pus, yet from the too evident necessity of the case, I urge the immediate extraction of the loose (at present) front teeth, to which the patient now readily consents.

The left lateral incisor adjoining the fistulous opening was affected in a peculiarly curious manner, viz. the fang was exostosed some distance from the apex, and of a blackened appearance. (Possibly the effect of the nit. sil.) The rest of the teeth were in a normal condition, so far as the substance of the tooth itself was concerned; but what was an indubitable evidence of the complete destruction of the bony snpport of the whole tooth, was the fact that the two central incisors had an attachment of muscle or fleshy deposit *the whole length* of their fangs; yet, upon extraction, the teeth parted from their *external* membrane without any unusual pain or difficulty, evidencing the previously mentioned deposit to have been *healthy fleshy granulations* to supply the loss by caries of the alveolar and jaw. I say *jaw*, because had there nothing been involved but the *alveolar support*, then these granulations would not have been found extending *to the very apex* of the root of the teeth.

Continued powders, arnica injection, and the chlo. pot. wash.

June 25th. Several spicula of bone have been working their way out since last visit, but gums appear in as healthy condition as could well be expected. The swelling in the palatine arch nearly subsided, with very little or no pain in the seat of the disease. The ridge of the jaw in front of the remaining molar, left side, is settling firm and sound, showing healthy absorption. From the fistulous opening (left canine tooth) there is still a slight discharge, but of a charaeter indicative of healthy action, of recuperation and supply.

So encouraging are the symptoms, that I have discharged the patient, with instructions to continue the arnica injection, being careful of his diet and habits, feeling assured that his troubles are thus brought to a favorable consummation. Advise him to have his loss supplied by an artificial denture, so soon as the condition of the jaw will admit, which he intends to do. Request him to call immediately should anything unfavorable transpire, and at any time when in town I request him to call and let me see how he is progressing.

July 20th. The patient is in excellent spirits and health, the "good bye" has been given to the pains of a lingering disease, and all things betoken a complete cure. The fistula is filling with healthy granulations, while the gums are closing properly in the front of the mouth, and this too without falling away so badly as I had reason to fear, on the loss of the teeth and the extended devastation wrought by the disease.

*Reflections.* In closing this article, which I fear has even now become more expanded than it ought, we may be pardoned in a few thoughts among many which naturally seem to present themselves as arising from a consideration of the above record.

We have here a case which in the short space of ten months passed beneath the judgment and notice of no less than four or five regular medical practitioners, all of whom pronounced the cure *certain* under the treatment by them prescribed, yet when the trial was made, the evident requirements of the case were not met.

Now was their ill success attributable to a false diagnosis, improper pathology, or unskillful therapeutics? Let us see.

In the earlier stages of the attack, what are the symptomatic and pathological indications, so far as we are enabled to deduce them from the expression of the patient—

evidently warranting to a hasty diagnosian the prognostic of the very complaint for which prescription was made, viz. local chemosis, or possibly, neuralgia facia? But may not the inquiry be opportune, was the diagnosis complete? and had it been, would there not have been predicated an entirely different treatment?

Again, in the second instance, in which case the M. D. was a surgeon and physician of considerable experience, having had a number of years practice in the Parisian hospitals, and also a successful practice in this country of eight or ten years, does it not appear strange, that with the way marked and seemingly apparent symptoms which the progress of the disease at this stage must have manifested, should betoken nothing more serious than what was pronounced "a chronic affection of the glands?"

That the teeth of the patient were all in an apparently healthy condition at the time of the first attack, I have no good reason to question, inasmuch as all the teeth remaining in the jaw when I first saw him were perfectly healthy so far as the substance of the tooth itself was concerned. The interrogation which immediately thrusts itself forward upon our mind, and one too, which is of particular moment to be discussed, is, what was the first predisposing cause or pathological derangement from which sprang the complication of diseases which we, in this case, have been called to meet and combat? Our own judgment strongly inclines to the following, as the only true and rational solution of the question.

That there was a decided idiosyncrasy strongly tending to the scorbutic, was too palpable to deny, which granted, it follows that from any predisposing or exciting cause which may have arisen, such as a violent cold, the antrum Highmoranium becoming the "locale" of the disorder, its membrane is inflamed; inflammation gives way to a purulent secretion, an engorgement and distention of the sinus follows, which, through erroneous diagnosis, is improperly treated, leading to a chronic affection, not only of the soft parts, but involving the more easily affected bones of the base of the sinus; abscess in turn succeeds, and this continues until by a *happy application* of mechanical skill the right incisor is extracted, thus affording egress to the pent up effusion, and a momentary relief. The pain now changes to a benumbing or throbbing, whereas before it was an acute, lancinating, darting *agony*. Not being properly treated, the ravages still progress, involving the subjacent parts; the teeth lose their support; the whole system sympathises with the parts diseased, and complication is the result.

